# Central Retinal Artery Occlusion: Can We Effectively Manage This Ocular Emergency in a Hospital Setting?

**DOI:** 10.7759/cureus.27840

**Published:** 2022-08-10

**Authors:** Maleesha Jayasinghe, Omesh Prathiraja, Abdul Mueez Alam Kayani, Rahul Jena, Malay Singhal, Minollie Suzanne Silva

**Affiliations:** 1 Medicine, Nanjing Medical University, Galle, LKA; 2 Medicine and Surgery, Nanjing Medical University, Melbourne, AUS; 3 Medicine and Surgery, Allama Iqbal Medical College, Lahore, PAK; 4 Medicine, Bharati Vidyapeeth Medical College/Bharati Hospital, Pune, IND; 5 Internal Medicine, Mahatma Gandhi Memorial Medical College, Indore, IND; 6 Medicine and Surgery, Nanjing Medical University, Nanjing, CHN

**Keywords:** ischemia, thrombolysis, treatment, retina, retinal vessels, ocular emergency, stroke, ophthalmological emergency, crao, central retinal artery occlusion

## Abstract

Central retinal artery occlusion (CRAO) is an ophthalmological emergency characterized by partial or complete occlusion of the central retinal artery. It is the ocular equivalent of an ischemic cerebral stroke. Patients frequently present with a significant, abrupt, painless loss of vision in one eye, with only around 20% of those affected getting functional visual acuity restored in the affected eye. Despite more than 150 years of clinical research, no consensus has been achieved regarding the most effective method of treating CRAO. The efficacy of all proposed treatments is debatable, and many of them have ambiguous risk profiles that present particular diagnostic and management difficulties and cause variations in clinical practice. In certain circumstances, thrombolysis may be attempted as a treatment option. However, the evidence to support the general use of thrombolytics in treating acute CRAO remains elusive. It is known that the risk factors predisposing to other cardiovascular and cerebrovascular events are often present in CRAO. Accordingly, identifying patients at the highest risk of stroke and secondary prevention of ischemic events remains the primary focus of management. This review offers a summary of all the current treatment options available for managing CRAO, with particular reference to their limitations and inconsistent results found in relevant studies until 2022.

## Introduction and background

The central retinal artery is a branch of the ophthalmic artery that supplies the macula and fovea of the inner retina. The thromboembolic occlusion of the central retinal artery is referred to as CRAO (central retinal artery occlusion). CRAO is a subtype of acute ischemic stroke with an estimated incidence of 1.8/100,000, reaching as high as 10/100,000 in people over 80 years of age. CRAO is clinically characterized by sudden, painless loss of vision in one eye, commonly described as a "curtain falling" or a generalized darkening. Less than 30% of patients experience spontaneous vision recovery, and prolonged monocular visual loss substantially impacts the quality of life. The duration of retinal ischemia is the most significant predictor of visual prognosis. Retinal ganglion cells suffer irreversible damage within 12-15 minutes of non-perfusion. Similar to an ischemic stroke, the period of permanent damage depends primarily on the extent of blockage and collaterals [1]. A study on rhesus monkeys revealed that in middle-aged or older atherosclerotic rhesus monkeys and those with arterial hypertension, central retinal artery blockage for less than 100 minutes showed no apparent morphometric evidence of optic nerve damage; nevertheless, CRAO of 105 minutes to less than 240 minutes created a varying degree of damage; and CRAO of 240 minutes or more induced complete or almost complete atrophy of the optic nerve and damage to nerve fibers. This signifies the existence of a therapeutic window in the management of CRAO [2].

Atherothrombosis at the lamina cribrosa is the leading cause of CRAO, accounting for around 80% of cases. Vasospasm and embolism are the other, albeit uncommon, causes of CRAO. CRAO, generally regarded as an ocular type of acute ischemic stroke, shares the same etiology, pathophysiology, and cardiovascular risk factors as ischemic stroke. To reduce the risk of ocular complications such as blindness and irreversible retinal damage, patients with CRAO should be evaluated immediately at a stroke center [3]. According to a study by Fallico et al., 30% of patients with acute CRAO and 25% of patients with acute branch retinal artery blockage exhibited MRI evidence of acute cerebral ischemia. Therefore, CRAO should not only be considered an ocular emergency but also a vascular event that may precede the development of life-threatening vascular complications, which warrants a care route consisting of the immediate referral of CRAO patients for neurologic examination and brain imaging [4].

Although the exact pathophysiology of CRAO is not yet fully established, several hypotheses have been proposed, mainly involving thromboembolic obstruction of the central retinal artery or, less frequently, vasospasm. As cardiovascular disease risk factors contribute to the pathogenesis of CRAO, they may induce endothelial dysfunction and early atherosclerosis by inducing a prothrombotic and pro-inflammatory state and decreasing vasodilation. In a case-control study involving 126 CRAO patients enrolled between 2013 and 2019, vascular endothelial damage, thicker intima-media thickness, and diastolic left ventricular cardiac dysfunction were seen, further establishing the link between CRAO and cardiovascular problems. In order to reduce the risk of future vascular complications in all CRAO patients, it is essential that we immediately adopt a systematic strategy and commence the necessary systemic treatment [3].

CRAO is a clinical diagnosis based on patient history and physical examination findings. The classic complaint involves a sudden, painless loss of vision from light perception to finger counting. Visual acuity may fall within the range of 20/200, but it is more frequently worse. If a patient experiences a lack of light perception, this could imply an obstruction of the ophthalmic artery and hence a lack of blood flow to the choroidal vessels. If central visual acuity is preserved and fundoscopy is consistent with CRAO, the patient likely has a cilioretinal artery blood supply. The ischemic retina may appear yellowish-white under fundoscopy. A "cherry-red spot" may occur on the macula; however, this is not a consistent finding. The presence of a cherry-red spot at the macula indicates a poor prognosis [5].

**Figure 1:**
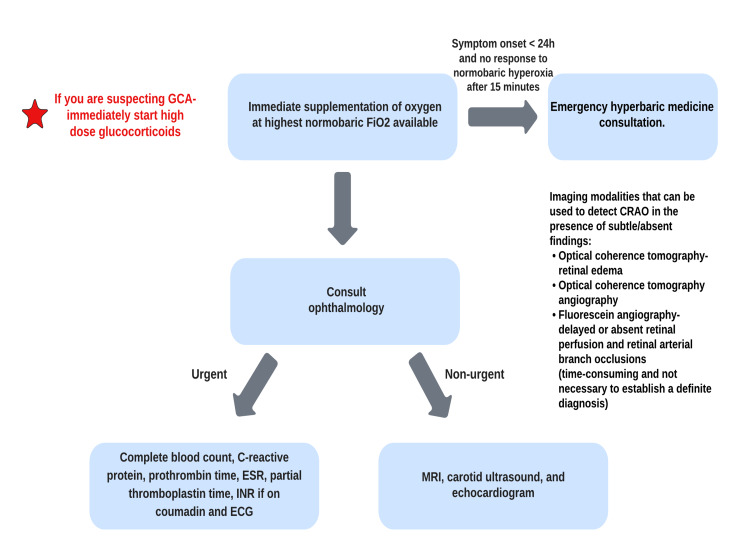
The general management of CRAO CRAO: central retinal artery occlusion; FiO_2_: fraction of inspired oxygen; GCA: giant cell arteritis; ESR: erythrocyte sedimentation rate; INR: international normalized ratio; ECG: electrocardiogram; MRI: magnetic resonance imaging Image credit: author Maleesha Jayasinghe

The general management of CRAO is illustrated in Figure 1.

Currently, there is no commonly accepted, evidence-based treatment for CRAO, in contrast to ischemic stroke, which has well-established guidelines for treatment. The fact that treatments for CRAO are rife with controversy reflects the desperation of treating physicians and contradictory findings in studies. Multiple factors, including insignificant collateral circulation at and distal to the usual site of CRAO, the small luminal diameter of the central retinal artery (0.12-0.17 mm), which makes it susceptible to occlusion, rapid infarction of central nervous tissues (ganglion cell infarction within 15 minutes), and embolic causes of CRAO that are resistant to thrombolysis, may explain why the treatment of non-arteritic CRAO remains problematic [6].

No one method of treatment for the management of CRAO has demonstrated efficacy to date. This could be attributable to the disease's obscure pathophysiology and its rarity in the population. Treatments such as hyperbaric oxygen therapy (HBOT) and intra-arterial tissue plasminogen activator (tPA) at early time points have shown promise, but further research is required. Some studies have found that HBOT therapy is most successful when administered within eight hours of the beginning of symptoms, while others have demonstrated that tPA administered after four hours from the onset of symptoms did not improve visual acuity due to irreversible damage to retinal structures. Additional research is required to better comprehend the precise time of retinal tissue death and the optimal timing for drug administration.

After discussing the benefits and hazards with the patient or surrogate, intravenous tPA may be chosen as an appropriate therapy option for people with CRAO. Since CRAO is a subtype of ischemic stroke, we must perform additional research and build systems of care for the urgent diagnosis, triage, and management of CRAO, similar to that for cerebral ischemic stroke. A combined effort involving a neurologist, an ophthalmologist, and an internist is essential in preventing the occurrence of CRAO in individuals with risk factors. Multicenter, randomized, double-blind, placebo-controlled clinical trials comparing intravenous tPA with placebo at initial time points in patients with CRAO are still lacking. Future studies should focus on creating novel indicators of retinal tissue viability accessible in real-time and complementing existing time-based decision-making algorithms, potentially permitting tPA administration at delayed time points in some patients.

Historical strategies (including anterior chamber paracentesis, ocular massage, and hemodilution) are not beneficial in terms of visual outcomes when used alone; however, when used in combination, they produce significant results. Thus, additional research involving different combinations of these drugs is required to identify the optimal combination treatment that can lead to favorable results when used in CRAO patients.

Surgery has achieved favorable outcomes for CRAO patients when combined with other pharmacological therapy. Therefore, it is essential to conduct clinical trials on CRAO patients by utilizing a combination of drugs and surgery to determine their efficacy. The evaluation of novel thrombolytic medications, such as tenecteplase, HBOT, and neuroprotectants in conjunction with recanalization therapy are additional treatment options that warrant further research.

In this review, we will discuss all existing treatments for CRAO and their effects on the prognosis of CRAO patients.

## Review

Study design

**Figure 2:**
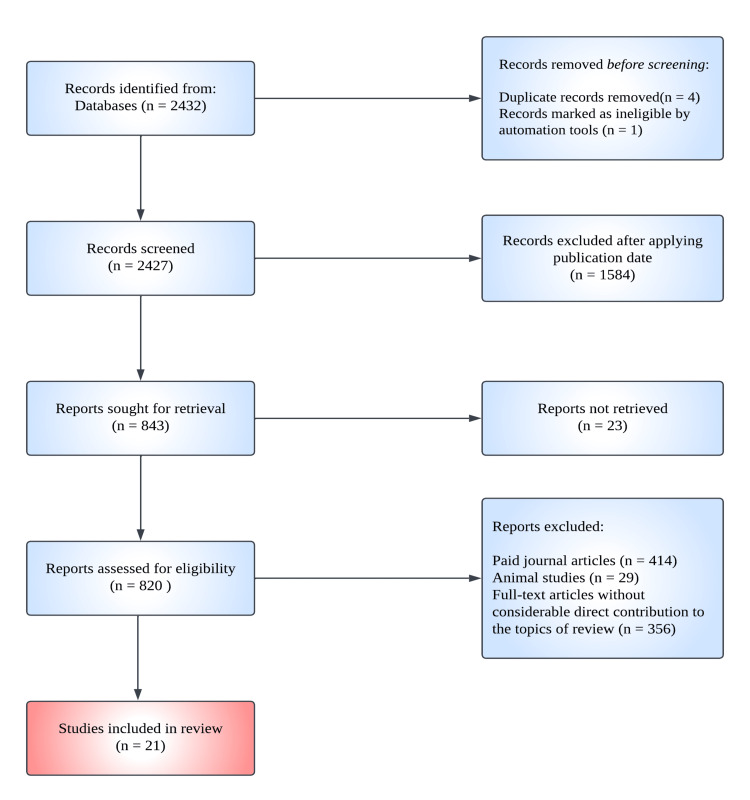
Selection process

This study aimed to review all published observational and clinical studies from January 1, 2015, to June 30, 2022, pertaining to therapeutic options used in the management of CRAO. In accordance with the PRISMA guidelines, a literature search was conducted using the online PubMed database and the following keywords: "central retinal artery occlusion," "treatment," and "management." The inclusion criteria included observational and clinical studies focusing on the various types of treatment. A preliminary search yielded 2,432 articles. Following the removal of all duplicate records and records deemed ineligible by automation tools, five articles were excluded. By limiting the "publication date" filter to articles published between 2015 and 2022, further 1,584 articles were eliminated. After examining the titles and abstracts, 799 articles that included paid full-text articles, animal studies, or full-text articles without a substantial direct contribution to the subject of study were eliminated, leaving 21 articles for this review. The selection procedure is illustrated in Figure 2.

Discussion

**Figure 3:**
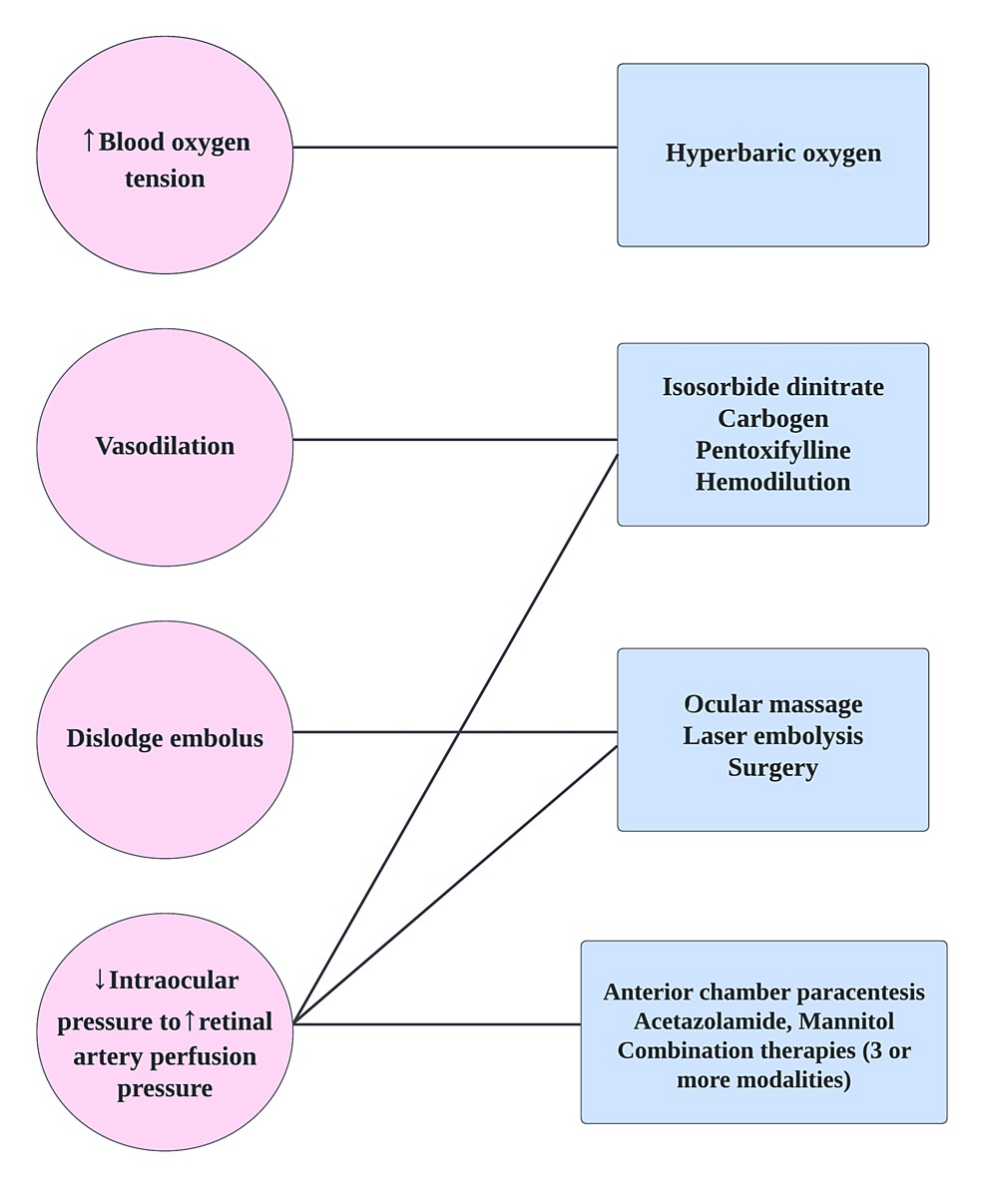
Pharmacological and non-pharmacological therapies for CRAO CRAO: central retinal artery occlusion Image credit: author Maleesha Jayasinghe

Below is a list of all the non-pharmacological and pharmacological methods utilized to treat CRAO. Figure 3 depicts all CRAO treatments and their mechanisms of action.

Non-pharmacologic Interventions

Hyperbaric oxygen therapy: HBOT entails the administration of 100% oxygen under atmospheric pressure two to three times that of normal air. During HBOT, the arterial and tissue oxygen pressure can reach a maximum of 2000 mmHg and 400 mmHg, respectively. At high pressure, the enhanced oxygen delivery to tissues can have a number of favorable effects on biochemical, molecular, and physiological processes, thereby promoting tissue healing. In the context of a retinal vascular obstruction, it is permissible to administer hyperbaric oxygen through medical compression chambers to preserve tissue life until reperfusion is achieved. Retinal artery constriction results in a considerable decrease in blood flow during the first 10 minutes of starting HBOT. This may be the result of the interplay between free oxygen radicals and nitric oxide that happens simultaneously with autoregulation during this treatment. As a result of the enhanced synthesis of nitric oxide immediately following the treatment's termination, rapid vasodilation occurs after the first 10 minutes of therapy. Although HBOT causes vasoconstriction of the retinal arteries, the increase in oxygen saturation is only 23%, and the retina is not harmed as a result. More specifically, vasoconstriction, which stops fluid leakage (fluid leakage causes retinopathy) and the improvement in macular and retinal oxygenation in regions with inadequate perfusion, a thickened basal membrane, or edema, are responsible for the beneficial effect of HBOT in the treatment of retinal vascular diseases [7].

Rozenberg et al. conducted a retrospective study in which 121 patients received HBOT while 23 patients only received standard of care (SOC). The medical records of patients diagnosed with non-arteritic CRAO without a patent cilioretinal artery in two tertiary medical centers were reviewed between January 2010 and December 2018. In the HBOT group, best-corrected visual acuity (BCVA) improved from 2.89 ± 0.98 logMAR at presentation to 2.15 ± 1.05 logMAR after HBOT, whereas there was no significant improvement in the SOC group. After accounting for age, gender, and duration of symptoms, the final BCVA of the HBOT group was significantly greater than that of the control group. The rates of patients achieving 20/200 or better vision were similar between the two groups [8]. Therefore, using HBOT as part of the SOC for CRAO improves the final visual outcome, making HBOT a safe method of treatment that can be implemented, if available, in all medical centers as part of the SOC.

Ocular massage: ocular massage (OM) is a technique designed to induce fluctuations in intraocular pressure (IOP) and facilitate the expulsion of the offending embolus. It is carried out using digital pressure or contact lenses. This enables retinal reperfusion by causing the embolus to disintegrate into a distal portion of the retinal vasculature. The OM technique involves repeatedly applying increased pressure to the globe for a period of 10-15 seconds, followed by "a sudden release with an in-and-out movement using a three-mirror contact lens for three to five minutes." Some authors have suggested massaging for up to 15-20 minutes [9]. OM is commonly performed on patients with CRAO, but there is no evidence that it is beneficial.

To our knowledge, no recent studies have demonstrated the efficacy of OM as a stand-alone treatment for CRAO. A prospective study in 2019 evaluated the effectiveness of OM in reducing IOP by causing a significant increase in retinal perfusion values. OM was performed for two minutes, with 10-second compression and decompression cycles. The study included 21 eyes from 21 participants with a median age of 29 years. IOP decreased significantly after OM, while subfoveal choroidal thickness, superficial capillary plexus perfusion (SCPP), deep capillary plexus perfusion, and choriocapillaris perfusion (CCP) increased significantly. Central macular thickness, radial peripapillary capillary perfusion, Sattler's layer perfusion, and Haller's layer perfusion exhibited no significant changes due to OM. Changes in SCPP were positively correlated with those in CCP and vice versa. It is necessary to conduct studies on patients with CRAO to understand the role of OM in CRAO, as this study was conducted on a healthy cohort of patients. However, these results suggest that OM is an effective treatment option for patients with CRAO [10].

Anterior chamber paracentesis: anterior chamber paracentesis is performed after the administration of a topical anesthetic and a prophylactic antibiotic (e.g., povidone-iodine). The cornea is then pierced with a 27-G needle attached to a tuberculin syringe or a paracentesis blade to drain a small amount of aqueous fluid. An operating microscope or slit lamp may be used to perform the procedure. A successful paracentesis should only remove the amount of aqueous fluid required to accomplish the desired IOP reduction. The normal anterior chamber volume is 250 μL, so the practitioner may remove small volumes (50 μL) at a time to achieve adequate IOP reduction while preserving the anterior chamber volume. Anterior chamber paracentesis rapidly reduces IOP and dilates retinal vessels, thereby increasing retinal perfusion pressure and promoting reperfusion of the retinal arterial system. These changes have been visualized using ocular coherence tomography (OCT) angiography of the retinal circulation [9]. Hwang et al. demonstrated a significant increase in macular perfusion in a 74-year-old patient using OCT angiography before and immediately after anterior chamber paracentesis. At presentation, OCT revealed acute inner retinal opacification. In contrast, it revealed partial preservation of inner retinal laminations 10 months later, which was attributed to reperfusion of superficial and deep retinal blood vessels [11].

Laser embolysis: in CRAO, photodisruption has been used to attempt physical breakdown (embolysis) or complete dislodgement (embolectomy) of emboli to restore retinal perfusion. Approximately 20% of CRAO cases show evidence of emboli. Neodymium-doped yttrium aluminum garnet (Nd:YAG) lasers have been predominantly used for this purpose. The procedure involves using a fundus contact lens, with the laser focused slightly posterior to the artery's wall at the embolus site. The power of the laser can be adjusted to achieve the desired effects [9]. Opremcak et al. administered translumenal Nd:YAG embolysis (TYL) or embolectomy (TYL/E) to 19 patients with vision-threatening CRAO or branch retinal artery occlusion and a visible embolus within the central retinal artery or branch retinal artery. The Nd:YAG laser was used with a fundus contact lens to target the embolus within the retinal arteriole. Laser treatment was delivered to the embolus with increasing energy until either the embolus fragmented within the lumen (embolysis) or passed into the vitreous through a small opening in the arteriole (embolectomy). Each of the 19 patients achieved TYL/E. In eight patients, the embolus was fragmented (embolysis), and in 11, it was transplaced into the vitreous (embolectomy). All patients exhibited reperfusion of the retina, as determined by fundus examination, fundus photography, and fluorescein angiography. Following reperfusion of the retina, there was an improvement in Snellen's visual acuity [12].

Surgical interventions: in the acute phase of CRAO, retinal endovascular surgery with tPA injection into the retinal artery improved visual acuity and retinal circulation. Takata et al. discovered that the visual acuity and retinal circulation of two patients with CRAO improved dramatically after undergoing retinal endovascular surgery with tPA injection into the retinal artery. A standard 25-G vitrectomy was performed in conjunction with the infusion of tPA into the retinal artery. The results of fluorescein angiography, laser speckle flowgraphy (LSFG), fundus photography, OCT, and changes in visual acuity were examined. Case one involved a 47-year-old female. One month following surgery, her visual acuity had significantly improved to 0.08 logMAR. However, OCT indicated retinal thinning in the macula. Case two involved a male aged 70 years. After two months of recovery following surgery, a rise in visual acuity from counting fingers to 0.1 logMAR was seen. In case two, LSG and fluorescein angiography revealed improved retinal circulation after surgery. This indicates that retinal endovascular surgery including the injection of tPA into the retinal artery is feasible and may enhance retinal circulation and, consequently, visual acuity in the acute phase of CRAO [13].

Another study revealed that vitreous surgery with direct massage of the central retinal artery appears to be an effective treatment for CRAO. Vitrectomy and direct central retinal artery massage were performed on 10 consecutive patients with acute CRAO. A special probe was used to massage the retinal artery inside the optic nerve, at the optic nerve head, or both, following a standard three-port pars plana vitrectomy. BCVA was assessed before surgery, and images of the fundus were taken before the procedure after one day, two days, and at weekly intervals for at least one month. Circulation was promptly restored in four cases during surgery, while it was gradually restored in four other cases starting the day after surgery. There was no change in the remaining two cases; however, one patient developed central retinal vein occlusion five days later. There were no other complications. At two months postoperatively, visual acuity improved in six cases (60%) by three or more lines, while it remained unchanged in the remaining cases [14].

Pharmacologic Interventions

IOP-lowering medications, sublingual isosorbide dinitrate, and pentoxifylline: IOP reduction is an intriguing potential therapeutic strategy because it is simple to use and has few side effects. Topical IOP-lowering drugs, such as intravenous mannitol, intravenous acetazolamide, and 50% oral glycerol, have been used to treat patients with acute CRAO. Topical medications are typically well tolerated and have various adverse effects that can be individualized for each patient. In contrast, oral and intravenous medicines like mannitol and acetazolamide are usually utilized to rapidly reduce IOP because topical treatments take a long time to accomplish the required IOP reduction. However, these drugs have many side effects, such as fatigue, paresthesias, and a metallic taste, in addition to potentially fatal complications, including Stevens-Johnson syndrome, allergy, metabolic acidosis, and blood pressure dyscrasias. Carbonic anhydrase inhibitors are frequently utilized in various ocular diseases, such as acute angle-closure glaucoma, despite their significant adverse effect profiles. As a result, most ophthalmologists are familiar with and comfortable with their use [9]. Unfortunately, there is insufficient evidence to support the use of IOP-lowering drugs in acute CRAO. Most studies are case reports or series that evaluate pharmacologic IOP lowering in conjunction with other treatments. None have established the benefits of lowering IOP as a stand-alone treatment.

Isosorbide dinitrate is a nitrate with vasodilator properties administered sublingually to treat angina. Due to its ability to synthesize nitric oxide, nitrates have been identified as contributors to the vascular tone of the choroid, optic nerve, and retina. The treatment's adverse effects include headache, vertigo, lightheadedness, and nausea. Isosorbide dinitrate has only been studied as a component of CRAO combination therapy. Isosorbide dinitrate has never been investigated as a stand-alone therapy. There is scant evidence beyond the fundamental scientific justification to support its usage as a therapy for CRAO [9].

The combination of sublingual isosorbide dinitrate, OM, intravenous mannitol or oral glycerol, intravenous acetazolamide, anterior chamber paracentesis, and intravenous methylprednisolone followed by streptokinase, and retrobulbar tolazoline improved visual acuity and retinal supply in 73% of patients suffering from unilateral CRAO with symptoms lasting less than 48 hours. All patients whose visual acuity improved had less than 12 hours of symptoms, and the presumed cause was either platelet-derived or cholesterol embolus from atheroma or glaucoma. Patients whose visual acuity did not improve had CRAO attributable to calcified emboli or primary antiphospholipid antibody syndrome, with symptoms occurring more than 12 hours before treatment. The success of the treatment in the systematic treatment group was better than the outcome in the nonsystematic treatment group, thereby corroborating the advantageous effects of combination therapy of pharmacological interventions in terms of CRAO treatment [15].

The phosphodiesterase inhibitor, pentoxifylline, is hypothesized to lower the viscosity of blood viscosity, red blood cell membrane rigidity, and the incidence of thrombus formation. For decades, it has been a potential treatment for retinal vascular disorders [9]. Ten patients with acute CRAO were evaluated in a randomized, controlled trial of oral pentoxifylline (1800 mg per day). Patients who received treatment had a more considerable increase in retinal blood flow as evaluated by duplex scanning and a more remarkable subjective visual improvement. However, no results regarding visual acuity were reported. Therefore, it cannot be considered that pentoxifylline treatment improved patients' vision [16]. Overall, the medicine is safe and well tolerated, posing only modest risks. Nevertheless, given the scarcity of evidence supporting its capacity to improve visual outcomes, routine administration of this drug in CRAO patients is not recommended [9].

Thrombolysis with tissue plasminogen activators: intravenous thrombolysis reduces the morbidity of acute ischemic stroke when administered within 4.5 hours of the last symptom-free interval. Currently, the American Heart Association's treatment guidelines for acute ischemic stroke do not address CRAO specifically. However, CRAO that causes retinal ischemia meets the criteria for an acute ischemic stroke (along with cerebral and spinal ischemia). The lack of treatment recommendation is likely due to a lack of high-quality randomized data demonstrating the benefit of thrombolysis in CRAO [17].

Kim et al. examined the angiographic parameters of the ophthalmic artery (OphA), ipsilateral carotid artery (CA), fundus photography, visual acuity, and fluorescein angiography in 101 patients with acute non-arteritic CRAO treated with intra-arterial thrombolysis (IAT). Out of 101 patients, 38 individuals had steno-occlusive lesions in the OphA, and 62 had atherosclerotic lesions in the ipsilateral CA. More severe plaque morphology was found in the ipsilateral CA in individuals who had a greater degree of occlusion in the OphA. Furthermore, even though these angiographic characteristics were unrelated to the visual outcome, the ipsilateral CA's lower degree of stenosis and less severe plaque morphology led to a notable increase in the eye's early reperfusion rate and arm-to-retina circulation following IAT. Overall, the study demonstrated a significant association between the severity of steno-occlusive lesions in the ipsilateral CA and the OphA in patients with CRAO. Patients with mild angiographic features in the CA had better retinal reperfusion after IAT, suggesting that angiographic characteristics in the CA could be a predictor of OphA vessel integrity and recanalization outcome after IAT [18].

Another randomized, placebo-controlled trial showed that intravenous tPA effectively treated eight patients with clinically defined CRAO within 24 hours of the onset of symptoms. tPA was administered at a total dose of 0.9 mg/kg, with 10% as a bolus over one minute and the remainder over one hour, and alterations in visual acuity were observed. Two out of eight patients in the tPA group experienced the primary outcome one week after receiving tPA, while no patients in the placebo group did. There was an intracranial hemorrhage in one patient. The improvement in visual acuity of these two patients did not persist at six months. In both cases, tPA was administered within six hours of the onset of symptoms. This randomized study adds to the evidence base for reperfusion in CRAO by demonstrating that the intervention window will likely be less than six hours. Reocclusion is a possible issue that may necessitate adjuvant anticoagulation. Future studies must focus on determining the efficacy of thrombolytics in the six-hour window [19].

A prospective randomized multicenter clinical trial in Europe that compared the treatment outcomes between conservative standard treatment (CST) and local intra-arterial fibrinolysis (LIF) for acute non-arteritic CRAO did not recommend LIF for the management of CRAO due to the high incidence of adverse reactions. In addition, the study found no statistically significant difference between the CST and LIF groups regarding the improvement in mean BCVA. Of note, 60% of patients treated with CST experienced clinically significant visual improvement, compared to 57.1% of patients treated with LIF. The data and safety monitoring committee recommended the termination of the study after the first interim analysis because 37.1% of patients treated with LIF experienced adverse reactions [20].

Some studies have shown that IAT is associated with glaucoma-related complications. Four of the six CRAO individuals treated with IAT, according to another retrospective analysis of the patients' medical records, went on to develop neovascular glaucoma. IAT was administered to all of these patients 4.5-6 hours after their first symptoms appeared. The patients did not have significant carotid artery stenosis, and their ophthalmic histories were unremarkable. Four of these patients developed excruciatingly painful neovascular glaucoma four to seven weeks after the onset of CRAO, even though no visual improvement was observed following treatment [21].

Limitations

Since our article focuses on a study of free full-text research journals published between 2004 and 2022, we may have omitted crucial information from paid full-text journals and research articles published before 2004. In addition, our analysis is confined to English-language studies, and hence we may have overlooked articles written in other languages.

## Conclusions

Current data mandates that CRAO should be treated with the same urgency as an acute ischemic cerebral stroke. It is a condition of the cerebral arterial tree that affects the retina, as well as an ophthalmological issue. There is a pressing need for randomized, controlled studies to investigate the numerous treatments available for intravenous thrombolysis during the therapy window of 4.5 hours. Treatments other than intra-arterial and intravenous thrombolysis have shown some improvement in the management of CRAO when provided as combination therapy. Therefore, it is crucial to conduct additional clinical trials on CRAO patients to identify the optimal combination of drugs required for optimal results in CRAO.

## References

[1] Ardila Jurado E, Sturm V, Brugger F (2022). Central retinal artery occlusion: current practice, awareness and prehospital delays in Switzerland. Front Neurol.

[2] Hayreh SS, Jonas JB (2000). Optic disk and retinal nerve fiber layer damage after transient central retinal artery occlusion: an experimental study in rhesus monkeys. Am J Ophthalmol.

[3] Dropiński J, Dziedzic R, Kubicka-Trząska A (2022). Central retinal artery occlusion is related to vascular endothelial injury and left ventricular diastolic dysfunction. J Clin Med.

[4] Fallico M, Lotery AJ, Longo A (2020). Risk of acute stroke in patients with retinal artery occlusion: a systematic review and meta-analysis. Eye (Lond).

[5] Hanley ME, Hendriksen S, Cooper JS (2022). Hyperbaric Treatment Of Central Retinal Artery Occlusion. https://www.ncbi.nlm.nih.gov/books/NBK431074/.

[6] Chronopoulos A, Schutz JS (2019). Central retinal artery occlusion-a new, provisional treatment approach. Surv Ophthalmol.

[7] Celebi AR (2021). Hyperbaric oxygen therapy for central retinal artery occlusion: patient selection and perspectives. Clin Ophthalmol.

[8] Rozenberg A, Hadad A, Peled A (2022). Hyperbaric oxygen treatment for non-arteritic central retinal artery occlusion retrospective comparative analysis from two tertiary medical centres. Eye (Lond).

[9] Sharma RA, Newman NJ, Biousse V (2021). Conservative treatments for acute nonarteritic central retinal artery occlusion: do they work?. Taiwan J Ophthalmol.

[10] Rommel F, Lüken S, Prasuhn M, Kurz M, Kakkassery V, Grisanti S, Ranjbar M (2020). Evaluating retinal and choroidal perfusion changes after ocular massage of healthy eyes using optical coherence tomography angiography. Medicina (Kaunas).

[11] Hwang CK, Kolomeyer AM, Brucker AJ (2017). Optical coherence tomography angiography of a central retinal artery occlusion before and after anterior chamber paracentesis. Ophthalmology.

[12] Opremcak E, Rehmar AJ, Ridenour CD, Borkowski LM, Kelley JK (2008). Restoration of retinal blood flow via translumenal Nd:YAG embolysis/embolectomy (TYL/E) for central and branch retinal artery occlusion. Retina.

[13] Takata Y, Nitta Y, Miyakoshi A, Hayashi A (2018). Retinal endovascular surgery with tissue plasminogen activator injection for central retinal artery occlusion. Case Rep Ophthalmol.

[14] Lu N, Wang NL, Wang GL, Li XW, Wang Y (2009). Vitreous surgery with direct central retinal artery massage for central retinal artery occlusion. Eye (Lond).

[15] Rumelt S, Dorenboim Y, Rehany U (2000). Aggressive systematic treatment for central retinal artery occlusion. Am J Ophthalmol.

[16] Incandela L, Cesarone MR, Belcaro G (2002). Treatment of vascular retinal disease with pentoxifylline: a controlled, randomized trial. Angiology.

[17] Mac Grory B, Lavin P, Kirshner H, Schrag M (2020). Thrombolytic therapy for acute central retinal artery occlusion. Stroke.

[18] Kim J, Jung S, Park KH, Woo SJ, Jung C (2022). Cerebral angiographic features of central retinal artery occlusion patients treated with intra-arterial thrombolysis. J Neurointerv Surg.

[19] Chen CS, Lee AW, Campbell B (2011). Efficacy of intravenous tissue-type plasminogen activator in central retinal artery occlusion: report from a randomized, controlled trial. Stroke.

[20] Schumacher M, Schmidt D, Jurklies B (2010). Central retinal artery occlusion: local intra-arterial fibrinolysis versus conservative treatment, a multicenter randomized trial. Ophthalmology.

[21] Furashova O, Thalwitzer J, Matthé E (2022). Early onset of neovascular glaucoma after intra-arterial thrombolysis for central retinal artery occlusion: a possible complication?. Clin Ophthalmol.

